# Supplementary material for: Major depressive disorder and suicide risk among adult outpatients at several general hospitals in a Chinese Han population
Source: PLoS One. 2017 Oct 10;12(10):e0186143. doi: 10.1371/journal.pone.0186143 (PMC5634639; doi:10.1371/journal.pone.0186143)
Supplement: S2 Checklist — (DOC) [file pone.0186143.s002.doc]

STROBE Statement—checklist of items that should be included in reports of observational studies

|  | Item No. | Recommendation | Page  No. | Relevant text from manuscript |
| --- | --- | --- | --- | --- |
| **Title and abstract** | 1 | (*a*) Indicate the study’s design with a commonly used term in the title or the abstract | 1 | Major depressive disorder and suicide risk among adult outpatients at several general hospitals in a Chinese Han population  Running title: major depressive disorder and suicide risk |
|  |  | (*b*) Provide in the abstract an informative and balanced summary of what was done and what was found | 2 | Abstract  Background: Somatic complaints are often the presenting symptoms of major depressive disorder (MDD) in the outpatient context, because this may go unrecognized. It is well understood that MDD carries an increased risk of suicide. This study aimed to identify the prevalence and risk factors for both MDD and suicidality among Han Chinese outpatients. Methods: A multicenter study was carried out in 5189 outpatient adults (≥18 years old) in four general hospitals in Guangzhou, China. The 1392 patients who had the Patient Health Questionnaire-9 (PHQ-9) score ≥ 5, indicating depressive symptoms were offered an interview with a psychiatrist by the Mini International Neuropsychiatric Interview (MINI); 819 patients consented and completed the MINI interview. MINI module B was used to assess suicidality. Stepwise binary logistic models were used to estimate the relationship between a significant risk factor and suicide or MDD. According to with or without MDD, the secondary analysis was performed using the logistic regression model for the risk of suicidility. Results: The lifetime prevalence of MDD and the one month prevalence of suicidality were 3.7% and 2.3% respectively. The odds ratio of suicidality in women was more than twice that in men (OR=2.62; 95% CI 1.45-4.76). Other risk factors which were significantly associated with suicidality were: living alone, higher education, self-reported depression, getting psychiatric diagnoses (MDD, anxiety disorders, and bipolar disorders). Significant risk factors for MDD were also noticed, such as comorbid anxiety disorders, self-reported anxiety, insomnia, suicidal ideation, and somatic complaints. Limitation: It’s a cross-sectional study in outpatient clinics using self-report questionnaires. Conclusion: This study provides valuable data about the prevalence of MDD, suicide risk, and their risk factors in adult outpatients in Han Chinese. Those factors allow better targeting of screening methods and the employment of preventative measures. |
| Introduction | | | |  |
| Background/rationale | 2 | Explain the scientific background and rationale for the investigation being reported | 3-4 | Major depressive disorder (MDD) is the most prevalent psychiatric disease and is a major contributor to the global burden of disease [1]. This burden originates in two major sequelae of depression: declining physical health, and suicide. MDD is responsible for 11% of years lived with a disability, and up to 15% of individuals with recurrent MDD commit suicide [2, 3]. Suicidality (ideation, plans, and attempts) is a significant cause of injury and mortality in the world, ranking as the 14th most common cause of death by the World Health Organization (WHO) [4, 5].  However, the prevalence of suicide is not homogenous. In China, it is the fifth most common cause of death and is the most common cause of death amongst adolescents and young adults (15-34 years old) [6]. This is in contrast to the United States, where suicide is the tenth most common cause of death and second most common cause of death amongst 15-34 years old [7]. Traditionally, research on suicide risk has been conducted mainly in Europe and the United States; however, the ability of a given risk factor to provoke suicidality clearly varies by region. Moreover, the prevalence of suicide and its associated risk factors have been under-investigated in China [8]. The city of Guangzhou provides a relevant setting within China to study risk factors for suicidality, as it is home to more than 13 million people and is highly cosmopolitan.  Psychiatric disorders are a well-established risk factor for suicidality [9]. Affective disorders possess the greatest risk; it has been estimated that half of all individuals who complete suicide meet criteria for MDD [8]. The association between MDD and suicidality is also very strong [10]. Thus, identifying MDD is a crucial component of any effective preventative screening for suicidality. However, given that half of patients who commit suicide do not meet MDD criteria, it is also important to screen for suicidality independently of an MDD diagnosis.  A nationwide study during 2004-2008 across China showed that 0.7% of Chinese adults had major depressive episode [11]. MDD has a point prevalence of 15% in the primary care setting, and 5% in the general population [12, 13]. Major depression is a disabling mood disorder that is present in 5–10% of primary care patients, including 10–20% of patients with chronic medical conditions [14]. Although there are effective interventions to reduce the burden of depression, the majority is inadequately treated [15]. The presence of depression is reported to worsen medical prognosis, increase physical symptom burden, complicate self-care and treatment-adherence, the costs of care, and probably also increase mortality [16, 17]. A number of population studies have shown an increased risk of associated with a range of specific physical illnesses [18, 19] .The need for more effective screening in both physical and mental health is evident. |
| Objectives | 3 | State specific objectives, including any prespecified hypotheses |  |  |
| Methods | | | |  |
| Study design | 4 | Present key elements of study design early in the paper | 4 | The aim of our study is twofold. First, we will to provide data on the prevalence of MDD, suicidality, and the risk factors which correlate with them in a Han Chinese population. Second, we will to identify an effective risk factor screening method for the general practitioner to use in the identification of both MDD and suicidality. |
| Setting | 5 | Describe the setting, locations, and relevant dates, including periods of recruitment, exposure, follow-up, and data collection | 4 | In this multicenter, cross-sectional study, data was collected from outpatient divisions in four tertiary general hospitals in Guangzhou from March 15 to June 30, 2016. The survey starting date was randomly selected. Three hospitals recruited participants from the gastroenterology, cardiology, and gynecology clinics; and all four hospitals recruited from their neurology clinics. |
| Participants | 6 | (*a*) *Cohort study*—Give the eligibility criteria, and the sources and methods of selection of participants. Describe methods of follow-up  *Case-control study*—Give the eligibility criteria, and the sources and methods of case ascertainment and control selection. Give the rationale for the choice of cases and controls  *Cross-sectional study*—Give the eligibility criteria, and the sources and methods of selection of participants | 4-5 | Participants were included if they were over 18 years old, consented to study participation, and were able to complete the PHQ-9 questionnaire. They were excluded if they did not or could not provide written informed consent, had previously received screening for depression, had a serious physical or mental condition, or had a language or hearing difficulty. In all, 5284 accepted the survey, but 5189 consecutive eligible adult outpatients completed the PHQ-9 questionnaire and were recruited for the statistics, finally, 819/1392 who got PHQ-9 scores≥5 received further psychiatric interview (see Fig1). |
|  |  | (*b*) *Cohort study*—For matched studies, give matching criteria and number of exposed and unexposed  *Case-control study*—For matched studies, give matching criteria and the number of controls per case |  |  |
| Variables | 7 | Clearly define all outcomes, exposures, predictors, potential confounders, and effect modifiers. Give diagnostic criteria, if applicable | 5 | First, the patient filled out a written questionnaire containing demographic questions and the PHQ-9 scale. Only those which were completed were considered valid and included in the study; then, the investigators administered the other self-report questionnaire. If the PHQ-9 score was greater than 4, the investigators referred the patient to one of the three psychiatrists. If the participant consented, the psychiatrist performed the structured MINI to confirm the presence of MDD met the DSM-Ⅳ diagnostic criteria and suicidality. |
| Data sources/ measurement | 8* | For each variable of interest, give sources of data and details of methods of assessment (measurement). Describe comparability of assessment methods if there is more than one group | 6-7 | 2.3 Research Instruments  2.3.1 Patient Health Questionnaire-9(PHQ-9) [20, 21]is a 9-item self-report questionnaire derived from the full PHQ. It assesses the presence of symptomatic criteria of a major depressive episode (MDE) and the severity of each symptom. It also evaluates frequency of each symptom via a 4-point Likert scale, from 0 (not at all) to 3 (nearly every day). Based upon the sum of these items, participants are categorized into 4 categories of depressive symptoms: minimal (0-4), mild (5-9), moderate (10-14), and severe (≥15).  2.3.2 Generalized Anxiety Disorder Scale-7(GAD-7) [22, 23]is a 7 question self-report questionnaire which assesses the frequency of symptoms of general anxiety via a 4-point Likert scale, from 0 (not at all) to 3 (nearly every day).  2.3.3 The 15-symptom Patient Health Questionnaire (PHQ-15) [24, 25] is 15-item self-report questionnaire derived from the full PHQ. It assesses the somatic symptoms and symptom clusters that account for more than 90% of physical complaints reported in the outpatient setting. Each item is scored on a 3-point Likert scale from 0 (not bothered at all) to 2 (bothered a lot) and the total is summed; a higher score indicates greater severity of somatic symptoms and physical complaints.  2.3.4 Medical Outcomes Study Short Form-12 (SF-12) [26, 27] is a 12-question self-report measure of patient health. It has two domains: the physical component score (PCS) and the mental component score (MCS). Higher scores indicate a better quality of life (QOL).  2.3.5 Insomnia is not measured in the SF-12, but is highly relevant to our study of depression. We assessed insomnia with 3 questions validated for screening in previous studies [28]. The questions were as follows: “In the past month, do you have difficulty falling asleep?”, “…do you have difficulty in maintaining sleep and in waking up?”, and “…do you wake up in the middle of the night or in the early morning and have difficulty falling asleep again?”. If at least one answer was “often”, then the patient was deemed to have insomnia.  2.3.6 Mini International Neuropsychiatric Interview 6.0.0 (MINI) [29, 30]is a psychiatrist-administered, structured interview assessing Diagnostic and Statistical Manual of Mental Diseases (DSM)-IV Axis I disorders. The MINI is robust and closely resembles the Structured Clinical Interview for the DSM-IV. The developer of the scale, Dr. David Sheehan, approved the use of the MINI 6.0.0 (Chinese version) for this investigation.  2.3.7 Mini International Neuropsychiatric Interview 6.0.0 module B (MINI module B) is a 15-question, yes-or-no interviewer-administered questionnaire used to evaluate suicidality and current risk of suicide. The first 3 questions screened for a recent suicide attempt in the past month, the next 14 for suicidal ideation, planning, or attempts in the past month, and the last question asks if the patient has ever attempted suicide in their life. The first 3 are excluded from the score, and the remainder are summed and categorized as low risk (<9), medium risk (9-16), or high risk (≥17) of future suicide attempts. |
| Bias | 9 | Describe any efforts to address potential sources of bias | 5 | All investigators participated in a two-day training session that described the clinical questionnaires, data collection methods, and goal of the study, as well as the ethical principles of human subject research. All questionnaires were administered by psychiatric postgraduates and senior students in clinical psychology. Three psychiatrists with more than 5 years of experience received four days of training on administering the Mini International Neuropsychiatric Interview (MINI) and used this tool to perform diagnostic interviews at each investigative site. |
| Study size | 10 | Explain how the study size was arrived at | 5 | In this multicenter, cross-sectional study, data was collected from outpatient divisions in four tertiary general hospitals in Guangzhou from March 15 to June 30, 2016. The survey starting date was randomly selected. Three hospitals recruited participants from the gastroenterology, cardiology, and gynecology clinics; and all four hospitals recruited from their neurology clinics.In all, 5284 accepted the survey, but 5189 consecutive eligible adult outpatients completed the PHQ-9 questionnaire and were recruited for the statistics, finally, 819/1392 who got PHQ-9 scores≥5 received further psychiatric interview (see Fig1). |

Continued on next page

| Quantitative variables | 11 | Explain how quantitative variables were handled in the analyses. If applicable, describe which groupings were chosen and why | 7-8 | Demographic data were reported as frequency or percentage. A Receiver Operating Characteristic (ROC) curve was employed to identify a continuous variable threshold for dichotomization of each of the aforementioned scales into “low” and “high” score categories (“low”: PCS ≤39; MCS ≤47). Patients were classified in two ways for analysis: those with vs those without MDD, and those with vs those without suicidality. The Chi-square or Fischer’s exact test was used to determine the statistical significance of differences in distribution of categorical sociodemographic and clinical risk factors between groups for a given classification. The independent sample t-test was used for continuous variables with approximately normal distribution. |
| --- | --- | --- | --- | --- |
| Statistical methods | 12 | (*a*) Describe all statistical methods, including those used to control for confounding | 8 | Stepwise binary logistic regression analysis with the forward conditional method was used to determine the correlates of MDD and suicidality. When MDD was used as the dependent variable, the independent variables were those factors which proved significantly different between participants with vs those without MDD in the bivariate correlation analysis. When suicidality was used as the dependent variable, the independent variables were those factors which proved significantly different between participants with vs those without suicidality in the bivariate correlation analysis. The level of significance was set at 0.05 (two-tailed). All statistical analyses were performed using SPSS21.0 statistical software (SPSS Inc., Chicago, United States). |
|  |  | (*b*) Describe any methods used to examine subgroups and interactions | 8 | The Chi-square test was used specifically for differences in groups based on marital status, living condition, and educational attainment. Comparison of the two groups’ rates with Bonferroni correction and the level of significance was 0.017. |
|  |  | (*c*) Explain how missing data were addressed | 7 | If an item was not reported, it was counted as a missing value. |
|  |  | (d) Cohort study—If applicable, explain how loss to follow-up was addressed  Case-control study—If applicable, explain how matching of cases and controls was addressed  Cross-sectional study—If applicable, describe analytical methods taking account of sampling strategy | 4 | In this multicenter, cross-sectional study, data was collected from outpatient divisions in four tertiary general hospitals in Guangzhou from March 15 to June 30, 2016. The survey starting date was randomly selected. |
|  |  | (e) Describe any sensitivity analyses |  |  |
| Results | | | | |
| Participants | 13* | (a) Report numbers of individuals at each stage of study—eg numbers potentially eligible, examined for eligibility, confirmed eligible, included in the study, completing follow-up, and analysed | 5 | In all, 5284 accepted the survey, but 5189 consecutive eligible adult outpatients completed the PHQ-9 questionnaire and were recruited for the statistics, finally, 819/1392 who got PHQ-9 scores≥5 received further psychiatric interview (see Fig1). |
|  |  | (b) Give reasons for non-participation at each stage | 5 | see Fig1. |
|  |  | (c) Consider use of a flow diagram | 5 | See Fig1. |
| Descriptive data | 14* | (a) Give characteristics of study participants (eg demographic, clinical, social) and information on exposures and potential confounders |  | Table 1 contains the summary of participant sociodemographic and clinical characteristics. The majority of the sample were women (66.2%), married (79.4%), and the plurality had medium educational attainment (7-12 years; 48.0%). 69.8% of participants reported low quality of life due to poor physical health and 45.3% reported low quality of life due to poor mental health on the SF-12 scale. 15.7% reported insomnia. The average scores on PHQ-9, GAD-7 and PHQ-15 scales were relatively 3.6±4.3, 3.2±4.2, and 5.0±4.5. There were 2.0% of participants with hopelessness. 89.2% of suicidal patients reported hopelessness, making it the most common symptom of suicidality. Suicide risk score stratification reported 1.6% of the sample at low, 0.5% at medium, and 0.2% at high risk of. The affective state evaluated by the MINI interview revealed a 3.5% current prevalence of anxiety spectrum disorders, a 1.6% current prevalence of bipolar disorder, a 3.7% MDD lifetime prevalence, and a 2.9% MDD prevalence within the past two weeks.  Suicidality was evaluated by suicide risk score on the MINI module B, with 2.3% of participants (120) reporting suicidal ideation within the past month and thus passing the screening phase. 0.2% (11) reported a suicide plan, and 0.1% (7) reported at least one prior suicide attempt; these are not mutually exclusive categories. Among the participants reporting suicidal ideation group, 55.8% had a lifetime prevalence of MDD; conversely, of the proportion of the sample reporting current MDD symptoms, 63.2% reported suicidal ideation, 5.8% a suicide plan, and 3.7% at least one prior suicide attempt (see Fig2). |
|  |  | (b) Indicate number of participants with missing data for each variable of interest | 7 | If an item was not reported, it was counted as a missing value. |
|  |  | (c) *Cohort study*—Summarise follow-up time (eg, average and total amount) |  |  |
| Outcome data | 15* | *Cohort study*—Report numbers of outcome events or summary measures over time |  |  |
|  |  | *Case-control study—*Report numbers in each exposure category, or summary measures of exposure |  |  |
|  |  | *Cross-sectional study—*Report numbers of outcome events or summary measures | 10-11 | Table 2 contains the results of bivariate correlation analysis of participant characteristics with either MDD or suicidality. We found 11 factors to be tentatively associated with lifetime MDD based on preliminary bivariate analyses. Patients with lifetime MDD were more likely to be  women, younger, unmarried. They were also more likely to report hopelessness, anxiety, insomnia, and physical complaints with a corresponding poor PCS and MCS. Living condition had statistic difference (χ2=7.309, p=0.026), but no significant difference between groups. There existed 14 factors associated with suicide risk. Patients with suicidality were more likely to be women, younger, unmarried, and to live alone and live in a nursing home or dormitory. They were also more likely to report depressed mood, anxiety, insomnia, and physical complaints with corresponding poor PCS and MCS. Both the suicidal group and MDD group were more likely to have comorbid psychiatric diagnoses. There was statistic difference in educational attainment (χ2=6.868, p=0.032), but no significant difference between groups. However, no group differed based on smoking rates, alcohol use, income, occupation, or whether they lived in urban or rural areas. Using the Chi-square test for differences in groups based on marital status, living condition, and educational attainment. In MDD, Never married was significantly different compared to the married (χ2=10.193, p=0.001). In suicide, Never married had significant difference, compared to the married (χ2=13.060, p＜0.001); both living alone and living in a nursing home or dormitory were significantly different, compared to live with families (χ2=10.382, p=0.001; χ2=7.211, p=0.007). There were no significant differences between the other groups. |
| Main results | 16 | (*a*) Give unadjusted estimates and, if applicable, confounder-adjusted estimates and their precision (eg, 95% confidence interval). Make clear which confounders were adjusted for and why they were included | 13 | Table 3 reports the results of the logistic regression model for MDD. Significant risk factors were: GAD-7 total score (OR=1.14 95% CI: 1.10-1.18), PHQ-15 total scores (OR=1.07 95% CI: 1.03-1.11), insomnia (OR=2.03 95% CI: 1.39-2.97), suicidal ideation (OR=7.50 95% CI: 4.50-12.48), and the presence of an anxiety disorder (OR=5.04 95% CI: 3.21-7.93). A high MCS score, reflective of quality of life in the mental realm, was protective against MDD (OR=0.49 95% CI: 0.33-0.74). Another 5 factors were significantly different in bivariate analyses, however, were not entered into regression model, including age, sex, marital, living alone, and poor PCS.  Table 3 also reports the results of the logistic regression model for suicidality. A diagnosis of MDD (OR=11.91 95% CI: 6.60-21.49), anxiety disorder (OR=4.39 95% CI: 2.53-7.61) and bipolar disorder (OR=7.94 95% CI 3.74-16.85) were significant risk factors. In addition, PHQ-9 total score was significant risk factor (OR=1.16 95% CI: 1.12-1.20). Three sociodemographic factors also proved significant: being female (OR=2.62 95% CI: 1.45-4.76), having medium educational attainment (7-12 years: OR=2.31 95% CI: 1.36-3.91), and living alone (OR=2.61 95% CI: 1.40-4.85). Another 7 factors proved not to be significant in the logistic regression model, including age, marital, insomnia, poor PCS & MCS, and PHQ-15 scores. |
|  |  | (b) Report category boundaries when continuous variables were categorized |  | A Receiver Operating Characteristic (ROC) curve was employed to identify a continuous variable threshold for dichotomization of each of the aforementioned scales into “low” and “high” score categories (“low”: PCS ≤39; MCS ≤47).the outcomes see Table 2,3&4. |
|  |  | (*c*) If relevant, consider translating estimates of relative risk into absolute risk for a meaningful time period |  |  |

Continued on next page

| Other analyses | 17 | Report other analyses done—eg analyses of subgroups and interactions, and sensitivity analyses | 13-14 | Furthermore, a secondary analysis was performed using the logistic regression model for the risk of suicidality in patients diagnosed with MDD. Significant risk factors were: female (OR=2.28 95% CI: 0.97-6.54), anxiety disorder (OR=2.24 95% CI: 1.17-4.28), other (living in a nursing home or dormitory; OR=3.45 95% CI: 1.18-10.14), and high PCS that was protective against MDD (OR=0.23 95% CI: 0.09-0.62).  A secondary analysis was also performed using the logistic regression model for the risk of suicidality in patients who did not receive a diagnosis of MDD. Significant risk factors were: Sex (OR=3.97 95% CI: 1.60-9.87), living alone(OR=2.89 95% CI: 1.27-6.57), Anxiety disorders (OR=8.45 95% CI: 3.76-18.99), Bipolar disorders (OR=4.42 95% CI: 1.93-10.16), GAD-7 total scores (OR=1.15 95% CI: 1.09-1.22) and PHQ-15 total scores (OR=1.09 95% CI: 1.03-1.15). |
| --- | --- | --- | --- | --- |
| Discussion | | | | |
| Key results | 18 | Summarise key results with reference to study objectives | 15-18 | Lifetime prevalence estimates of MDD vary widely across the globe and range from 3% (Japan) to 16.9% (United States) [31]. The global 12-month prevalence of MDD has recently been estimated at 4.7-6.7% [9, 32]. Several recent epidemiological studies of mental disorders in China (1.2-8.6%), and a survey of 50 general hospital internal medicine clinics in China reported lower levels of MDD prevalence (3.95%) as compared to the global average [33]. Our study found a 3.7% lifetime prevalence of MDD, which is in keeping with reported levels in.  Lifetime prevalence of suicidal ideation also varies widely; one study found that, across 9 countries, this varied from 2.09% to 18.51% in the general population [34]. Another survey of 17 countries found a 9.2% lifetime prevalence of suicidal ideation [4]. In western countries, past-year suicidal ideation ranges from 2.4% to 6.9% [35]. In comparison, a survey of 2877 outpatients over 15 years of age at general hospitals in Beijing found a lifetime prevalence of suicidal ideation at 6.08% [36]. However, another study of 5926 outpatients in Beijing comparing urban to rural inhabitants found lower levels of lifetime suicidal ideation (1.8% urban, 2.8% rural), indicating significant variation in this statistics that may depend upon interviewing techniques, sampling bias, and the rapid socioeconomic change which many cities in China are experiencing [37]. We did not evaluate lifetime prevalence; instead, we found a 2.3% past-month prevalence rate which is surprisingly high, given that this is equivalent to the lifetime prevalence found in the aforementioned study in Beijing. Another study showed 4.8% of inpatients with suicidal ideation in past one month were higher than ours [38].  Our evaluation of the risk factors for MDD was consistent with literature both within China and globally: being female, being unmarried, and being younger [2, 39]. Our report of significant risk factors for suicidality was in line with many other cross-national studies in China: being female, unmarried, living alone, being younger, not attaining college education [4, 35, 40]. Being female is a unique risk factor in China; in most countries, being male is a strong risk factor and being female is considered protective against suicide [34]. Multiple hypotheses have been put forward without strong confirmation or refutation. A most recent Ethiopian study showed that women had 63% higher odds of endorsing suicidal risk than men at general hospital (OR=1.63 95%CI: 1.13–2.36) [9]. The Hong Kong survey also showed that female gender was associated with a higher risk of suicidal ideation [41]. The Chinese national reports on suicide showed a strong reversal of the international trend: in those younger than 60 years, female rates exceed male rates by an average of 26% [8]. While women also reported significantly higher rates of MDD than men in our study, this is consistent both within China and globally, and does not explain the difference in suicidality. Women may report MDD more commonly than men for multiple reasons: social inequality and oppression, higher rates of sexual and physical abuse, higher healthcare utilization and willingness to report symptoms, and the presence of female-specific affective disorders due to fluctuation in estrogen and progesterone levels such as premenstrual dysphoric disorder [8, 42, 43].  MDD is well-known as a primary risk factor for suicidality, and this was borne out in our study (OR=11.91; 95% CI: 6.60-21.49). The prevalence of suicidal ideation among our participants with MDD (55.4%) was consistent with the literature, which reports rates from 47% to 69% [44, 45]. A nationwide study showed that 15% of 0.5 million Chinese adults with major depressive episode had suicidal ideation [11]. Hopelessness is another major risk factor for suicidality, as described by Britton et al.; we found that 89.2% of patients reporting suicidality also reported hopelessness and this was significantly more common than patients without suicidality [46]. There is a sequence of suicidality; as the situation persists, a person moves from suicidal ideation, to developing a plan, and then to attempting suicide within the first year after onset of suicidal ideation [10]. There is a limited window in which to prevent this outcome; this fact encourages the employment of more sensitive and more rigorous screening and more intense intervention in order to reduce suffering and death. This is particularly true within China, where rates of utilization of psychiatric services for suicidal ideation are low. One study found that, in Beijing, 36.2% of suicidal subjects sought medical help for their suicidality, and only 20.7% consulted a psychiatrist [37].  Anxiety and depression are commonly comorbid and may represent different facets of a single underlying mental phenomena; they are more commonly comorbid than separate [47]. Anxiety in the context of MDD increases the likelihood of suicidal ideation [48]. This has been demonstrated in Chinese patients as well [49]. Indeed, anxiety has been found as an independent risk factor for suicidal ideation and suicide attempts [50]. Our study corroborates this; the presence of anxiety in patients with MDD was a significant risk factor for suicidality (OR=2.24 95% CI: 1.17-4.28), as well as in all patients reporting suicidality (OR=4.39 95% CI: 2.53-7.61).  The secondary logistic regression analysis suggested us that one patient with MDD had some characteristics such as female, living in a nursing home or dormitory, and combine anxiety disorders, so they will increase her suicide risk. When we see the patients without MDD in general hospitals, however, they are female and live alone, self-report anxious mood and somatic complaints, getting anxiety disorders and bipolar disorders, we should be alert her who are more likely to have suicide risk. |
| Limitations | 19 | Discuss limitations of the study, taking into account sources of potential bias or imprecision. Discuss both direction and magnitude of any potential bias | 18 | The major strengths of our study were the large sample size, the use of reliable and cross-validated clinical diagnostic assessments, and the significant results it produced. However, there are several limitations to this study. First, because it is cross-sectional, no causality can be assumed between the significant risk factors and MDD or suicidality. Second, because all data was reliant upon self-report, it is subject to recall bias, response bias, and a desire for non-disclosure because of the sensitive nature of the material at hand. |
| Interpretation | 20 | Give a cautious overall interpretation of results considering objectives, limitations, multiplicity of analyses, results from similar studies, and other relevant evidence | 19 | 6. Conclusions  Our results demonstrate that MDD and suicidality are both common among adult outpatients with somatic complaints. Chinese-specific risk factors for suicide were verified and expounded upon, with women who are unmarried, of higher education, living alone, self-report depression or anxiety，and have psychiatric diagnoses being at much greater risk of suicidal ideation. Significant risk factors for MDD were also detailed, such as comorbid anxiety, self-reported anxiety, insomnia, suicidal ideation, and somatic complaints. Given the low utilization of psychiatric services in China, our data provides a strong impetus for non-psychiatric clinicians consulting a patient with somatic complaints to screen for, elaborate upon, and treat or refer depression and suicidality to a psychiatrist. Improving health may resolve depression in some patients, but others may require appropriate referral and direct psychiatric care [51]. Our data also displays the need for strengthening mental health promotion, anti-stigma campaigns, prevention programs, public education about the availability of treatment for depression, anxiety, and suicidality, and early intervention at populations at high risk of MDD and suicide [8]. |
| Generalisability | 21 | Discuss the generalisability (external validity) of the study results | 18 | the clinical context of non-psychiatric outpatient clinics within urban general hospitals may have induced some selection bias, and limits generalizability to the broader Chinese population. |
| Other information | |  | | |
| Funding | 22 | Give the source of funding and the role of the funders for the present study and, if applicable, for the original study on which the present article is based | 19-20 | National Key Research and Development Plan "Precision Medical Research" 2016 Project 2016YFC0906302; Guangzhou Medical and Health Science and Technology Major Projects 20151A031003; We acknowledgement support from grant from the National Science and Technologic Program of China 2015BAI13B02. |

*Give information separately for cases and controls in case-control studies and, if applicable, for exposed and unexposed groups in cohort and cross-sectional studies.

**Note:** An Explanation and Elaboration article discusses each checklist item and gives methodological background and published examples of transparent reporting. The STROBE checklist is best used in conjunction with this article (freely available on the Web sites of PLoS Medicine at http://www.plosmedicine.org/, Annals of Internal Medicine at http://www.annals.org/, and Epidemiology at http://www.epidem.com/). Information on the STROBE Initiative is available at www.strobe-statement.org.
