# Supplementary figures and images for: Major depressive disorder and suicide risk among adult outpatients at several general hospitals in a Chinese Han population
Source: PLoS One. 2017 Oct 10;12(10):e0186143. doi: 10.1371/journal.pone.0186143 (PMC5634639; doi:10.1371/journal.pone.0186143)

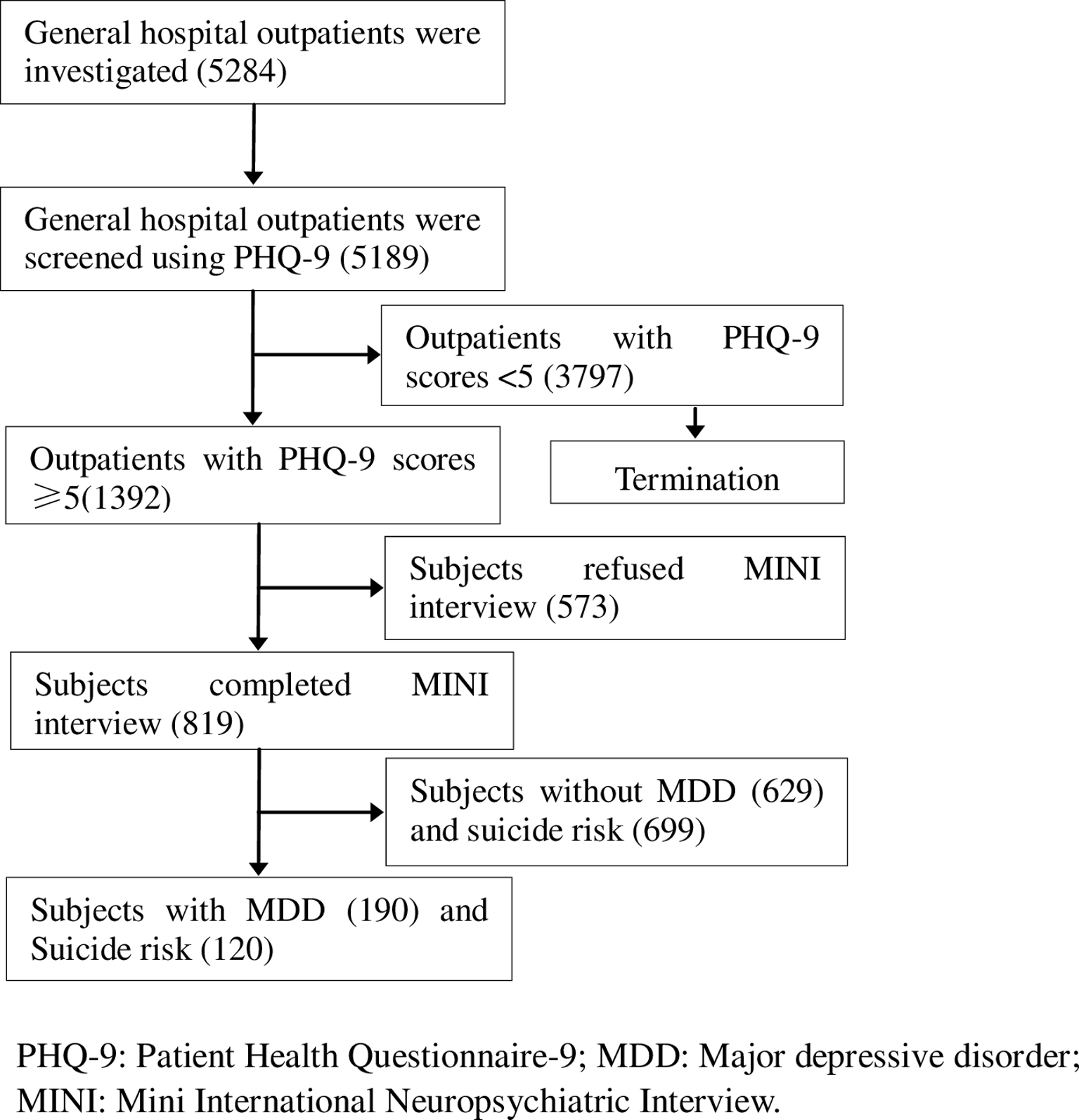

Supplement: S1 Fig — Flowchart of the study on prevalence of major depressive disorders and suicide risk from general hospitals in Guangzhou. (TIF) [file pone.0186143.s004.tif]

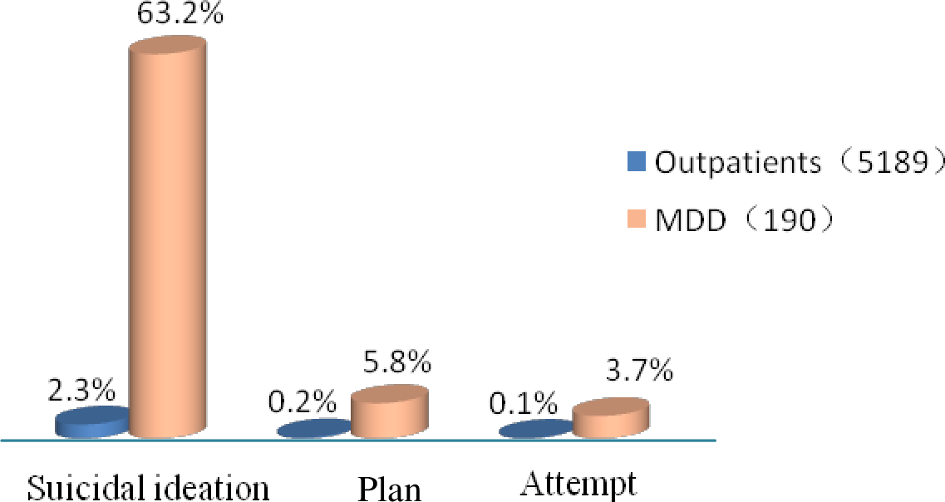

Supplement: S2 Fig — (TIF) [file pone.0186143.s005.tif]
