# Supplementary material for: Major depressive disorder and suicide risk among adult outpatients at several general hospitals in a Chinese Han population
Source: PLoS One. 2017 Oct 10;12(10):e0186143. doi: 10.1371/journal.pone.0186143 (PMC5634639; doi:10.1371/journal.pone.0186143)
Supplement: S1 Table — (DOCX) [file pone.0186143.s006.docx]

**Table 1. Characteristics of the Demographic and Clinical Characteristics of the study population** (N=5189).

| Characteristics | N | % |
| --- | --- | --- |
| Sex |  |  |
| Male | 1753 | 33.8 |
| Female | 3436 | 66.2 |
| Education |  |  |
| Illiterate or primary school (0-6) | 992 | 19.1 |
| Junior and senior high school(7-12) | 2490 | 48.0 |
| College and above(≥13) | 1707 | 32.9 |
| Marital status |  |  |
| Never married | 807 | 15.6 |
| Married | 4122 | 79.4 |
| Other(divorce/widowed) | 260 | 5.0 |
| Living condition |  |  |
| Alone | 468 | 9.0 |
| Live with families | 4354 | 83.9 |
| Other^a^ | 367 | 7.1 |
| PCS |  |  |
| High(＞39) | 1568 | 30.2 |
| Low(≤39) | 3621 | 69.8 |
| MCS |  |  |
| High(＞47) | 2840 | 54.7 |
| Low(≤47) | 2349 | 45.3 |
| Smoking(yes) | 584 | 13.2 |
| Drinking(yes) | 970 | 21.6 |
| Any insomnia(yes) | 979 | 18.9 |
| Major depressive disorder(yes) | 190 | 3.7 |
| Anxiety disorders(yes) | 174 | 3.4 |
| Bipolar disorders(yes) | 85 | 1.6 |
| Hopelessness(yes) | 107 | 2.0 |
|  | Mean±SD ^b^ |  |
| Age(years) | 42.1±16.0 |  |
| PHQ-9 total scores | 3.6±4.3 |  |
| GAD-7 total scores | 3.2±4.2 |  |
| PHQ-15 total scores | 5.0±4.5 |  |

^a^ Other (living in a nursing home or dormitory);

^b^ Continuous variables, Mean± standard deviation (SD);

120 patients had suicide risk, of which there are 84 low scores (1-8), moderate to 26 individuals (9-16), high score of 10 people (≥17).

PHQ-15: Patient Health Questionnaire somatic symptom severity scale-15.

PCS: physical component score of SF-12; MCS: mental component score of SF-12;

PHQ-9: Patient Health Questionnaire-9; GAD-7: Generalized Anxiety Disorder Scale-7.
