# Supplementary material for: Major depressive disorder and suicide risk among adult outpatients at several general hospitals in a Chinese Han population
Source: PLoS One. 2017 Oct 10;12(10):e0186143. doi: 10.1371/journal.pone.0186143 (PMC5634639; doi:10.1371/journal.pone.0186143)
Supplement: S2 Table — (DOCX) [file pone.0186143.s007.docx]

**Table 2. Demographic and Clinical Characteristics of subjects according to Major depressive disorder and Suicide risk (N=5189).**

| **Characteristics** | **MDD** | **Non-MDD** | **χ2** |  | **Suicidality** | **Non-suicidality** |  | ***p-value*** |
| --- | --- | --- | --- | --- | --- | --- | --- | --- |
|  | **N=190** | **N=4999** |  | ***p-value*** | **N=120** | **N=5069** | **χ2** |  |
|  | **n(%)** | **n(%)** |  |  | **n(%)** | **n(%)** |  |  |
| Sex |  |  | 5.642 | **0.019** |  |  | 19.809 | **＜0.001** |
| Female | 141(4.1) | 3294(95.9) |  |  | 103(3.0) | 3332(97.0) |  |  |
| Male | 49(2.8) | 1704(97.2) |  |  | 18(1.0%) | 1735(99.0) |  |  |
| Education |  |  | 1.004 | 0.605 |  |  | 6.868 | **0.032** |
| Illiterate or primary school | 31(3.1) | 961(96.9) |  |  | 16(1.6) | 976(98.4) |  |  |
| Junior and senior high school | 94(3.8) | 2396(96.2) |  |  | 72(2.9) | 2418(97.1) |  |  |
| College and above(≥13) | 65(3.8) | 1642(96.3) |  |  | 33(1.9) | 1674(98.1) |  |  |
| Marital status |  |  | 13.342 | **0.001** |  |  | 13.076 | **0.001** |
| Never married | 44(5.5) | 763(94.5) |  |  | 33(4.1) | 774(95.9) |  |  |
| Married | 131(3.2) | 3991(96.8) |  |  | 82(2.0) | 4040(98.0) |  |  |
| Other(divorce/widowed) | 15(5.8) | 245(94.2) |  |  | 6(2.3) | 254(97.7) |  |  |
| Living condition |  |  | 7.309 | **0.026** |  |  | 15.144 | **0.001** |
| Alone | 25(5.3) | 443(94.7) |  |  | 20(4.3) | 448(95.7) |  |  |
| Live with families | 146(3.4) | 4208(96.6) |  |  | 86(2.0) | 4268(98.0) |  |  |
| Other ^a^ | 19(5.2) | 348(94.8) |  |  | 15(4.1) | 352(95.9) |  |  |
| PCS |  |  | 9.765 | **0.002** |  |  | 11.009 | **0.001** |
| High(＞39) | 38(2.4) | 1530(97.6) |  |  | 20(1.3) | 1548(98.7) |  |  |
| Low(≤39) | 152(4.2) | 3469(95.8) |  |  | 101(2.8) | 3520(97.2) |  |  |
| MCS |  |  | 82.020 | **＜0.001** |  |  | 44.816 | **＜0.001** |
| High(＞47) | 43(1.5) | 2797(98.5) |  |  | 30(1.1) | 2810(98.9) |  |  |
| Low(≤47) | 147(6.3) | 2202(93.7) |  |  | 91(3.9) | 2258(96.1) |  |  |
| Smoking(yes) | 18(3.1) | 566(96.9) | 1.219 | 0.303 | 11(1.9) | 573(98.1) | 0.810 | 0.368 |
| Drinking(yes) | 35(3.6) | 935(96.4) | 0.404 | 0.525 | 21(2.2) | 949(97.8) | 0.355 | 0.551 |
| Any insomnia(yes) | 99(10.1) | 880(89.9) | 142.344 | **＜0.001** | 60(6.1) | 919(93.9) | 76.379 | **＜0.001** |
| Anxiety disorders(yes) | 82(47.1) | 92(52.9) | 964.204 | **＜0.001** | 62(35.6) | 112(64.4) | 876.606 | **＜0.001** |
| Suicidal ideation(yes) | 67(55.4) | 54(44.6) | 939.112 | **＜0.001** | - | - | - | - |
| Bipolar disorders(yes) | - | - | - | - | 20(23.5) | 65(76.5) | 170.494 | **＜0.001** |
| Major depressive disorder(yes) | - | - | - | - | 67(35.3) | 123(64.7) | 939.112 | **＜0.001** |
|  |  |  |  |  |  |  |  |  |
|  | **‾x±s** | **‾x±s** | **t** | ***p-value*** | **‾x±s** | **‾x±s** | **t** | ***p-value*** |
| Age(years) | 38.1±12.97 | 38.1±12.97 | -3.476 | **0.001** | 35.9±11.7 | 42.2±16.0 | -4.318 | **＜0.001** |
| PHQ-9 total scores | - | - | - | - | 13.0±5.9 | 3.4±4.0 | 25.877 | **＜0.001** |
| GAD-7 total scores | 10.9±5.8 | 2.9±3.9 | 27.245 | **＜0.001** | 11.3±5.8 | 3.0±4.0 | 22.285 | **＜0.001** |
| PHQ-15 total scores | 11.7±5.3 | 5.3±4.2 | 19.962 | **＜0.001** | 12.7±5.7 | 5.4±4.2 | 17.960 | **＜0.001** |

^a^ Other (living in a nursing home or dormitory).

PHQ-9: Patient Health Questionnaire-9; GAD-7: Generalized Anxiety Disorder Scale-7;

PCS: physical component score of SF-12; MCS: mental component score of SF-12;

PHQ-15: Patient Health Questionnaire somatic symptom severity scale-15.
