# Supplementary material for: Major depressive disorder and suicide risk among adult outpatients at several general hospitals in a Chinese Han population
Source: PLoS One. 2017 Oct 10;12(10):e0186143. doi: 10.1371/journal.pone.0186143 (PMC5634639; doi:10.1371/journal.pone.0186143)
Supplement: S3 Table — (DOCX) [file pone.0186143.s008.docx]

**Table 3. Logistic regression results for factors associated with Major depressive disorder and Suicide risk.**

| **Variable** | **Major depressive disorder** | | | |
| --- | --- | --- | --- | --- |
|  | **OR** | **95%CI** | | ***p*-value** |
| Anxiety disorders(Yes vs. No) | 5.0 | 3.2-7.9 | | **＜0.001** |
| Insomnia(Yes vs. No) | 2.0 | 1.4-3.0 | | **＜0.001** |
| MCS(High vs. Low) | 0.5 | 0.3-0.7 | | **0.001** |
| Suicidal ideation (Yes vs. No) | 7.5 | 4.5-12.5 | | **＜0.001** |
| PHQ-15 total scores | 1.1 | 1.0-1.1 | | **＜0.001** |
| GAD-7 total scores | 1.1 | 1.1-1.2 | | **＜0.001** |
|  | **Suicide risk** | | | |
|  | **OR** | | **95%CI** | ***p-value*** |
| Sex(Female vs. Male) | 2.6 | | 1.5-4.8 | **0.001** |
| Major depressive disorder(Yes vs. No) | 11.9 | | 6.6-21.5 | **＜0.001** |
| Anxiety disorders(Yes vs. No) | 4.4 | | 2.5-7.6 | **＜0.001** |
| Bipolar disorders(Yes vs. No) | 7.9 | | 3.7-16.9 | **＜0.001** |
| PHQ-9 total scores | 1.2 | | 1.1-1.2 | **＜0.001** |
| Education(vs. College and above(≥13) |  | |  | **0.005** |
| Illiterate or primary school(0-6) | 1.3 | | 0.6-2.8 | 0.506 |
| Junior and senior high school(7-12) | 2.3 | | 1.4-3.9 | **0.002** |
| Living condition(vs. Live with families) |  | |  | **0.005** |
| Alone | 2.6 | | 1.4-4.9 | **0.002** |
| Others ^a^ | 1.9 | | 0.9-3.9 | 0.095 |

^a^ Other (living in a nursing home or dormitory).

GAD-7: Generalized Anxiety Disorder Scale-7;

PHQ-15: Patient Health Questionnaire somatic symptom severity scale-15.

PHQ-9: Patient Health Questionnaire-9; MCS: mental component score of SF-12;
