# Supplementary material for: Major depressive disorder and suicide risk among adult outpatients at several general hospitals in a Chinese Han population
Source: PLoS One. 2017 Oct 10;12(10):e0186143. doi: 10.1371/journal.pone.0186143 (PMC5634639; doi:10.1371/journal.pone.0186143)
Supplement: S4 Table — (DOCX) [file pone.0186143.s009.docx]

**Table 4. The secondary logistic regression results for factors associated with suicide risk**

| **Variable** | **With major depressive disorder** | | | |
| --- | --- | --- | --- | --- |
|  | **OR** | **95%CI** | | ***p-value*** |
| Sex(Female vs. Male) | 2.3 | 1.0-5.1 | | **0.042** |
| Anxiety disorders(Yes vs. No) | 2.2 | 1.2-4.3 | | **0.015** |
| PCS(High vs. Low) | 0.2 | 0.1-0.6 | | **0.004** |
| Living condition(vs. Live with families) |  |  | | **0.027** |
| Alone | 2.5 | 1.0-6.5 | | 0.057 |
| Others ^a^ | 3.5 | 1.2-10.1 | | **0.024** |
|  | **Without major depressive disorder** | | | |
|  | **OR** | **95%CI** | ***p-value*** | |
| Sex(Female vs. Male) | 4.0 | 1.6-9.9 | **0.003** | |
| Anxiety disorders(Yes vs. No) | 8.5 | 3.8-19.0 | **＜0.001** | |
| Bipolar disorders(Yes vs. No) | 4.4 | 1.9-10.2 | **＜0.001** | |
| GAD-7 total scores | 1.2 | 1.1-1.2 | **＜0.001** | |
| PHQ-15 total scores | 1.1 | 1.0-1.2 | **0.004** | |
| Living condition(vs. Live with families) |  |  | **0.041** | |
| Alone | 2.9 | 1.3-6.6 | **0.012** | |
| Others ^a^ | 1.4 | 0.5-4.1 | 0.557 | |

^a^ Other (living in a nursing home or dormitory).

PHQ-15: Patient Health Questionnaire somatic symptom severity scale-15.

PCS: physical component score of SF-12; GAD-7: Generalized Anxiety Disorder Scale-7;
