# Supplementary material for: Major depressive disorder and suicide risk among adult outpatients at several general hospitals in a Chinese Han population
Source: PLoS One. 2017 Oct 10;12(10):e0186143. doi: 10.1371/journal.pone.0186143 (PMC5634639; doi:10.1371/journal.pone.0186143)
Supplement: S1 File — (PDF) [file pone.0186143.s010.pdf]

广州市惠爱医院伦理委员会

审 批 件

（2016）第（021）号

研究项目名称	广州市综合医院抑郁焦虑障碍患者早期筛查和全程干预模式的研究
申办单位	广州市惠爱医院
主要研究者及地址	宁玉萍　　广州市荔湾区明心路 36 号，邮编：510370
审阅及批准的文件	1、研究方案<br>2、知情同意书

表决意见

同意	修改后同意	不同意	终止或暂停	弃权	请假
[illegible]	0	0	0	0	2

审批结论

1. 委员会对该方案的审查决定如下(在口内划×)
   - ☒同意
   - 口修改后同意
   - 口不同意
   - 口终止或暂停试验
2. 该研究进行过程中将接受伦理委员会的持续审查? 口是 ☒否

   审查频率：口 3 个月　口 6 个月　口 12 个月

主任委员：[illegible]

签名日期：2016.12.29

（印章：广州市惠爱医院伦理委员会）

附件	委员名单
声明	本伦理委员会的职责、人员组成、操作程序及记录遵循 ICH-GCP、中国的相关法律法规。<br>This ethics committee’s responsibilities, composition, function, operations and records are fully compliant with ICH-GCP, and related regulation and law of China.<br>本批件自签字日期起一年内有效，过期请重新提交申请。

（广州市惠爱医院伦理委员会地址：广州市荔湾区明心路 36 号）
