# Supplementary material for: Major depressive disorder and suicide risk among adult outpatients at several general hospitals in a Chinese Han population
Source: PLoS One. 2017 Oct 10;12(10):e0186143. doi: 10.1371/journal.pone.0186143 (PMC5634639; doi:10.1371/journal.pone.0186143)
Supplement: S2 File — (DOCX) [file pone.0186143.s011.docx]

**知情同意书**

**知情页**

**尊敬的受访者：**

我们邀请您参加广州市综合医院抑郁焦虑障碍患者早期筛查和全程干预模式的研究。该研究是广州市医药卫生科技重大项目，由广州市卫生和计划生育委员会资助。在您参加研究前，请仔细阅读下面的信息。如果您有不清楚的地方或您想了解更多的信息，请向我们咨询。

**[研究目的]**

本次调查的目的是了解广州市综合医院患者的抑郁焦虑障碍患病情况，获得综合医院抑郁焦虑障碍患者临床特征、患病率及分布情况的基础数据，形成适用于广州地区综合医院的早期筛查和个体化的全程干预模式及防治模型，为卫生行政部门完善精神障碍患者的筛查、治疗转介等政策法规的制定提供基础数据。

**[研究过程概述]**

本研究需要对受访者进行情绪状态以及社会心理等相关评估，对部分受访者将进行血液学及颅脑影像学检查。这些评估和检查都是免费的。如果您符合随访的入选标准，您将被随机分配到药物治疗组、心理治疗组、药物治疗合并心理治疗组或自然观察对照组，观察期为期一年，于基线期、2周、4周、12周、24周及48周各接受一次评估和检查。研究过程中，请您按医生要求的时间到医院就诊或接受电话访谈。

**[获益与风险]**

参加本项研究，您的病情可能获得改善，通过本临床观察所得到的信息可能会有助于进一步改进针对此病的治疗方法，这可能会给病情和您类似的其他患者带来好处。

**[参与原则]**

参与该项研究完全是自愿的，您和您的监护人可以自愿决定是否参加，在研究中的任何时候您都可以退出。您的医疗待遇或合法利益不会因此受到任何影响，但希望您能配合直至观察结束；研究人员出于对您的最大利益考虑，可能会随时中止/终止您参加本项观察。如果您需要退出观察，为了您的安全和客观评价药物的作用，请您能配合主管医生完成观察结束后的相关评价和实验室检查。我们将为您提供适当的误工、误餐及交通补贴。

**[研究内容知晓权]**

您的身份等隐私资料将严格保密，除研究者外，任何第三方都不会知道您的身份等隐私资料，您参加研究意味着允许研究者使用研究获得的信息。

**签名页**

在参加“广州市综合医院抑郁焦虑障碍患者早期筛查和全程干预模式的研究”之前，我声明：

我已被告知本项研究的研究目的、研究内容与方法，我已充分了解该研究的性质、意义、可能带来的风险及益处。我拥有自愿参加或拒绝参加本研究的权利。我的个人信息资料将得到保密。

我完全理解了上述内容，经过充分的考虑，自主作出决定：我自愿参加本次研究，作为本研究的一名受试者，履行受试者的权利和义务，与研究医生合作，按要求参与有关检查或随访。

受访者签名：

签名（正楷） 签名 日期： 年 月 日

联系电话：

法定监护人如适用请签名，**□**适用 **□**不适用

法定监护人签名：

签名（正楷） 签名 日期： 年 月 日

联系电话：

**研究者宣言，我或我的研究人员已向受试者充分解释了本研究的情况和参加本研究所带来的益处和风险。他的签名是有效的。医学问题、语言或教育程度不妨碍对上述情况的了解。**

研究者签名：

签名（正楷）： 签名 日期： 年 月 日
