# Supplementary material for: Major depressive disorder and suicide risk among adult outpatients at several general hospitals in a Chinese Han population
Source: PLoS One. 2017 Oct 10;12(10):e0186143. doi: 10.1371/journal.pone.0186143 (PMC5634639; doi:10.1371/journal.pone.0186143)
Supplement: S3 File — (DOC) [file pone.0186143.s012.doc]

Professors **Joerg Heber**

Editors-in-Chief, ***PLOS ONE***

Aug 21, 2017

Dear Professors Heber,

Please find enclosed our manuscript entitled *“Major depressive disorder and suicide risk among adult outpatients at several general hospitals in a Chinese Han population”* by Hai-Yan Li et al. for consideration of publication as an Original Report in your respected journal *PLOS ONE.*

Increasing evidence suggests that Somatic complaints are often the presenting symptoms of major depressive disorder (MDD) in the outpatient context and may go unrecognized. This burden originates from two major sequelae of depression: declining physical health, and suicide. However, to our knowledge, research on suicide risk has been conducted mainly in Europe and the United States; the ability of a given risk factors to provoke suicidality clearly varies by region. Moreover, the risk factors of suicide and MDD have been under-investigated in China. In this study, we found the odds ratio of suicidality in women was more than twice that in men (OR=2.6; 95% CI 1.45-4.76). Other risk factors that were significantly associated with suicidality were the following: living alone, higher education, self-reported depression, and psychiatric diagnoses (MDD, anxiety disorders, and bipolar disorders). Significant risk factors for MDD were also reported, such as comorbid anxiety disorders, self-reported anxiety, insomnia, and suicidal ideation.

The submission has had all of the authors’ approval. The work presented here has not been published previously, nor is it being considered for publication elsewhere. All clinical study described in the manuscript were carried out in accordance with Declaration of Helsinki promulgated by the National Institute of Health. I have read and have abided by the statement of ethical standards for manuscripts submitted to PLOS ONE. All authors declare no conflict of interest. We hope that this manuscript would be suitable for publication in the PLOS ONE.

I will act as corresponding author and can be contacted directly via the numbers below. I hope that the reviewing process finds the manuscript favorable for publication in your respected journal.

This study was funded through National Key Research and Development Plan "Precision Medical Research" 2016 Project (2016YFC0906302); Guangzhou Medical and Health Science and Technology Major Projects (20151A031003); the National Science and Technologic Program of China(2015BAI13B02). The funders had a role in study design, data collection and analysis, decision to publish, or preparation of the manuscript.

I look forward to any future correspondence. Thank you very much in advance for all the trouble you’ll take. Your kind assistance is greatly appreciated.

Yours sincerely,

Yu Ping Ning, MD, PhD.

Southern Medical University, China.

Guangzhou Medical University Affiliated Brain Hospital, Guangzhou Huiai Hospital, China.

36 Mingxin Road, Liwan District, Guangzhou 510370, Guangdong, China.

Tel: 86-13922214239 Email: [ningjeny@126.com](mailto:ningjeny@126.com)
